# Familial “benign” pemphigus? Erythroderma and fatal outcome^☆^^☆☆^

**DOI:** 10.1016/j.abd.2019.02.006

**Published:** 2019-11-25

**Authors:** Paula Baldissera Tansini, Ana Letícia Boff, Magda Blessmann Weber, Renan Rangel Bonamigo

**Affiliations:** a Public Health Dermatology Service, Secretaria Estadual de Saúde, Porto Alegre, RS, Brazil; b Instituto de Dermatologia Prof. Rubem David Azulay, Rio de Janeiro, RJ, Brazil; c Dermatology Service, Santa Casa de Misericórdia de Porto Alegre, Porto Alegre, RS, Brazil; d Dermatology Service, Universidade Federal de Ciências da Saúde, Porto Alegre, RS, Brazil; e School of Medicine, Universidade Federal do Rio Grande do Sul, Porto Alegre, RS, Brazil

**Keywords:** Pemphigus, benign familial, Dermatitis, exfoliative, Skin diseases, vesiculobullous

## Abstract

Hailey–Hailey disease, or familial benign pemphigus, is a rare bullous genodermatosis that usually presents with flaccid blisters, erosions, and maceration limited to flexural areas, resulting in increased morbidity and reduced quality of life for affected patients. The authors report an unusual case of generalized Hailey–Hailey disease with erythroderma and fatal outcome.

## Introduction

Hailey–Hailey disease is a rare cutaneous disorder, featuring dominant autosomal inheritance with complete penetrance and variable expressivity, affecting adhesion between the epidermal keratinocytes. It is caused by a mutation in the ATP2C1 gene, which encodes the intracellular calcium pump, altering the homeostasis of this ion. Calcium is involved in cellular differentiation, skin barrier repair, cellular adhesion, and keratinocyte motility. Its increased levels lead to the alteration of desmosomes and cellular adhesion to the epidermal junction, causing acantholysis. Characterized by painful blistering, erosions, and maceration, it primarily involves the flexural areas in a symmetric fashion.^1^

## Case report

**Figure 1:**
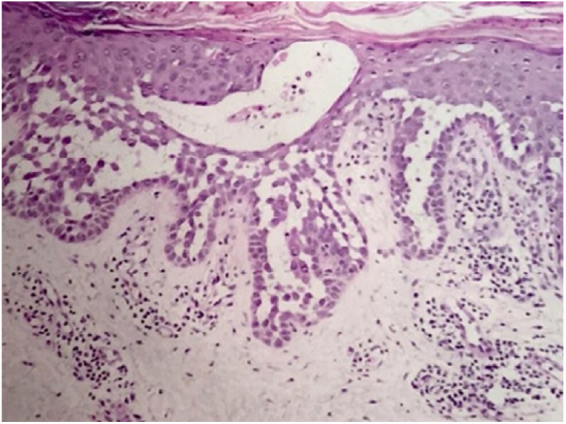
Extensive suprabasal acantholysis with intraepidermal bullae and keratinocytes with “dilapidated brick wall” appearance (Hematoxylin & eosin, ×40).

**Figure 2:**
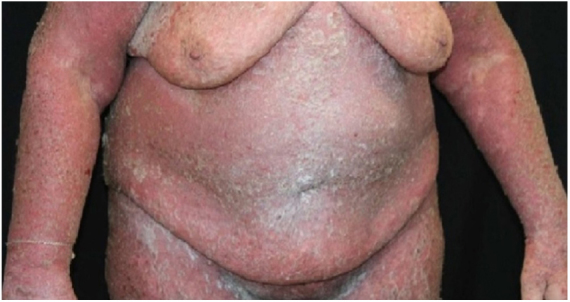
Exfoliative erythroderma. Intertriginous areas with extensive erythema and tendency to maceration.

**Figure 3:**
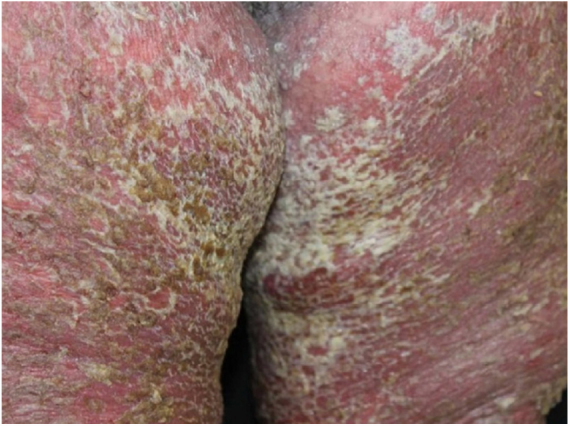
Diffuse erythema, vesicular lesions, erosions, and crusts in lower limbs close to inguinal folds.

A 64-year-old woman presented with erythematous plaques with peripheral detachment and multiple erosions in the inframammary, axillary, inguinal, dorsal, and cubital and popliteal fossa regions. Mucous membranes were unaffected. She reported a family history of mother, siblings, and maternal aunts with similar lesions, and previous medical history of systemic hypertension, coronary artery disease, congestive heart failure, dyslipidemia, and hypothyroidism. Histopathological examination revealed acantholysis with intra-epidermal cleavage involving the entire epidermal thickness, demonstrating absence of dermal villi and eosinophils (Fig. 1). The patient was diagnosed with Hailey–Hailey disease based on clinical, familial, and histopathological data. Given the multiple comorbidities of the patient, treatment was limited to tetracycline, prednisone, dapsone, sulfamethoxazole + trimethoprim, cephalexin, and methotrexate, with little or no response. After one year of follow-up, the patient developed erythroderma (Figure 2, Figure 3) and systemic symptoms, and was hospitalized. She died due to venous catheter-related infectious complications one month after hospitalization.

## Discussion

Hailey–Hailey disease was first described in 1939 by the Hailey brothers.^1^ Its prevalence remains unknown and its autosomal dominant inheritance pattern shows complete penetrance and variable expressivity. Its pathogenesis involves ATP2C1 gene mutation, leading to changes in calcium transport into the Golgi apparatus that result in desmosomal dysfunction and keratinocyte acantholysis.^2^

Disease progression is characterized by chronicity and periods of exacerbation, with onset usually occurring in the second and third decades of life. Clinically, flaccid vesicles and erosions are present, with erythematous, occasionally macerated plaques that symmetrically involve the flexural, axillary, inframammary, inguinal, and cervical regions. The segmental form is usually unilateral and linear (along the lines of Blaschko).^3^ The generalized form, as in the case reported here, is rare, and a common complication is secondary infection by fungi and bacteria. Other factors associated with worsening of symptoms include heat, sweating, friction, and ultraviolet radiation.^4^ The main differential diagnoses are pemphigus vulgaris, Darier's disease, inverse psoriasis, seborrheic dermatitis, intertrigo, and erythrasma.^5^ The diagnosis is confirmed by histopathology, which shows extensive suprabasal acantholysis with “dilapidated brick wall” appearance and, possibly, dyskeratotic keratinocytes. Immunofluorescence is classically negative in Hailey–Hailey disease, being unnecessary in the presence of typical clinical and histopathological features.^3^ Treatment options are limited and depend on clinical severity. They include oral and topical antibiotics, topical calcineurin inhibitors, oral and topical corticosteroids, dapsone, acitretin, methotrexate, and cyclosporine.^6^ Treatment alternatives for recalcitrant cases are botulinum toxin, surgical resection, laser therapy, and photodynamic therapy.^7^, ^8^ In most cases, clinical symptoms are limited to flexural and intertriginous areas. Generalized manifestations are uncommon, and erythroderma with fatal outcome is extremely rare. The few cases reported in the literature describe bacterial infection,^9^ cutaneous adverse drug reaction, and herpes^10^ as triggering factors for an acute dissemination of the disease. However, these factors differ from those of the case reported here, since the patient presented with chronic generalized involvement, regardless of the diagnosis of secondary infections and antibiotic therapies, and no triggers for erythroderma were identified.

This was, therefore, an extremely severe case of Hailey–Hailey disease that was highly resistant to treatment, in a patient with multiple comorbidities, resulting in the death of the patient.

## Financial support

None declared.

## Authors’ contribution

Paula Baldissera Tansini: Composition of the manuscript.

Ana Letícia Boff: Intellectual participation in the propaedeutic and/or therapeutic conduct of the studied cases.

Magda Blessmann Weber: Intellectual participation in the propaedeutic and/or therapeutic conduct of the studied cases.

Renan Rangel Bonamigo: Approval of the final version of the manuscript; intellectual participation in the propaedeutic and/or therapeutic conduct of the studied cases; critical review of the manuscript.

## Conflicts of interest

None declared.
